# The Effect of Interpersonal Relationship and Social Activity on the Physical and Mental Health of Older Korean Adults

**DOI:** 10.1093/geroni/igab046.3334

**Published:** 2021-12-17

**Authors:** Meeryoung Kim, Linda Park

**Affiliations:** 1 Daegu University, Daegu, Kyongsang-bukto, Republic of Korea; 2 University of Wisconsin-Madison, Madison, Wisconsin, United States

## Abstract

Maintaining interpersonal relationships and social activities are important as you get older. Activity theory indicates that social activities and human relations are important factors for older adults’ physical and mental health. However, the effects between the quantity and quality of interpersonal relationships and social activities will be different. This study compared which of the effects has a greater impact between interpersonal and social activities on physical and mental health. This study used the 6th additional wave (2016) and 7th wave (2017) of the Korean Retirement and Income Study. The subjects of this study were older adults who are aged 65 and older and the sample size was 2,152. Multiple regression was used for data analysis. Demographic variables were controlled. Independent variables were interpersonal relationships, social activities, satisfaction with interpersonal relationships, and satisfaction with social activities. Dependent variables were physical health and mental health, with depressive symptoms used as a proxy for mental health. βs was used to determine the relative influence on dependent variables. Interpersonal relationships, satisfaction with interpersonal relationships, and satisfaction with social activities significantly influenced physical health. Among them, interpersonal satisfaction was found to be the most influential factor on physical health. In addition, interpersonal satisfaction was found to be the most influential factor on mental health than interpersonal relationships. Satisfaction with social activities only affected physical health. The implications of this study were that the quality of interpersonal relationships and social activities of older adults affected physical and mental health more than quantity.

